# An Efficient Distributed Approach for Cooperative Spectrum Sensing in Varying Interests Cognitive Radio Networks

**DOI:** 10.3390/s22176692

**Published:** 2022-09-04

**Authors:** Maria Trigka, Elias Dritsas

**Affiliations:** Department of Computer Engineering and Informatics, University of Patras, 26504 Patras, Greece

**Keywords:** cognitive radio, spectrum sensing, diffusion strategies, adapt-then-combine, cooperation

## Abstract

The rapid growth in wireless communications, coupled with insufficient utilization of the spectrum, led to the development of new wireless services and the promising technology of cognitive radio (CR) networks, which facilitate periodic access to the unoccupied spectrum bands and thus increases spectral efficiency. A fundamental task in CR networks is spectrum sensing, through which unauthorized secondary users (SUs) detect unoccupied bands in the spectrum. To achieve this, an accurate estimate of the power spectrum is necessary. From this perspective, and given that many other factors can affect individual detection, such as pathloss and receiver uncertainty, we aim to improve its estimate by exploiting the spatial diversity in the SUs’ observations. Spectrum sensing is treated as a parameters estimation problem, assuming that the parameters’ vector of each SU consists of some global and partially common parameters. To exploit this modeling, distributed and cooperative spectrum sensing is the subject of interest in this study. Diffusion techniques, and especially the Adapt-Then-Combine (ATC) method will be exploited, where each SU cooperates with a group of nodes in its neighborhood that share the same parameters of interest. We consider a network of three static PUs with overlapping power spectrums, and thus, frequency bands. The performance of the employed method will be evaluated under two scenarios: (i) when the PUs spectrum varies, since some frequency bands are not yet utilized, and (ii) when the frequency bands of the PUs are fixed, but there is a mobile SU in the network, changing regions and parameters of interest. Experimental results and performance analysis reveal the ATC algorithm robustness and efficiency.

## 1. Introduction

The radio frequency spectrum is an inherently limited resource. Many services provided to users every day are based on the broadcast of radio signals (radio, television, telephony, broadband internet, defense applications (e.g., radar), scientific uses such as meteorology, positioning (GPS), etc.). New technologies, such as the Internet of Things and Machine-to-Machine communication, appear that rely on the use of wireless radio systems (UMTS, Wi-Fi, WiMax, Digital TV, 5G, and B5G). Additionally, the transition to multimedia applications, and in general, the rapid growth in wireless communications have contributed to a great demand for the development of new wireless services for both the authorized and unauthorized frequency spectrums [1].

At the same time, the electromagnetic spectrum, because of greater and greater increases in demand, is in short supply. Most of it is occupied by already existing services and applications. Therefore, to assign a part of the electromagnetic spectrum to a new service, an existing one should be replaced, cease its operation, and thus free the requested spectrum band. However, the electromagnetic spectrum will still be fully occupied. On the other hand, a significant part of the already allocated spectrum has limited or sporadic usage, thus leading to its underuse.

All of the above create the requirement for continuously available spectrum frequencies without interference. Recent studies have shown that the current fixed spectrum assignment policy has imposed insufficient use of the spectrum. In recent years, dynamic spectrum management in 5G networks is more necessary, due to the increasing demand for free frequencies solving the problem of spectral inadequacy [2]. To address this problem, Cognitive Radio Networks have been brought to the foreground as a promising technology that facilitates periodic access to unoccupied frequency bands, contributing to the increase in spectrum efficiency [3].

According to the Federal Communications Commission (FCC), cognitive radio is a system that senses its operating electromagnetic environment and can dynamically and autonomously adjust its radio operating parameters to modify the operation of the system, such as to maximize the throughput, mitigate interference, facilitate interoperability and access the secondary networks. Hence, one main purpose of cognitive radio is the autonomous exploitation of the locally unused part of the spectrum (spectrum holes) with the aim of providing new access paths to it [4].

The main functions of the cognitive radio networks (spectrum sensing, management, mobility, sharing) are represented in a cycle called the cognitive radio cycle [5]. Cognitive Networks manage the problem of congestion frequencies by introducing the occasional use of frequency bands that are not occupied by authorized users and are known as spectrum holes. The CR network users can share the spectrum with primary users. Primary users have higher priority or authorization in the use of an available piece of the spectrum, and use the service to which the specific bandwidth is assigned, thus maximizing the degree of spectrum utilization [6]. Secondary users of the cognitive network have a lower priority and exploit the available spectrum in such a way that they do not create interference with primary users. The fundamental work of each of the SUs is to detect and identify the presence or absence of authorized users, known as primary users. It is usually achieved by sensing the RF environment through a process called spectrum sensing. The way of measuring and exploiting the spectral space is called spectrum coverage, which is stated as being a frequency band that is not used by its PU, at a specific time, in a specific geographic area [7]. CR networks, along with device-to-device communication, are two key technologies for efficient spectrum sharing, especially in next-generation networks [8,9].

In the current study, we will be concerned with cooperative sensing, and especially distributed learning and distributed estimation methods about intelligent wireless sensor networks similar to [10]. Specifically, our aim is to estimate the total spectrum of three PUs with overlapping frequency bands. Capitalizing on the relevant literature, the problem has been previously studied for either independent [11] or overlapping spectrums among two PUs [12]. Additionally, the main contribution of the presented analysis is the evaluation of the ATC diffusion-based method in more challenging and practical scenarios, where some frequency bands stop being utilized by some PUs, or where there is a mobile SU node in the network. The experimental results verify the efficiency of the method in such challenging conditions.

The rest of the paper is organized as follows. Section 2 describes the relevant spectrum sensing works with the subject under consideration. Furthermore, in Section 3, an analysis of the system model and the adopted methodology followed are made. In addition, in Section 4, we discuss the acquired research results. Finally, conclusions and future directions are outlined in Section 5.

The following notations are considered in this paper. Uppercase and lowercase bold letters denote matrices and vectors, respectively. ${\left(\cdot \right)}^{T}$ and ${\left(\cdot \right)}^{H}$ denote the transposition and the complex conjugate transposition of a matrix, I is the identity matrix, $\lambda_{\max}\left(\cdot \right)$ is the maximum eigenvalue of a matrix, ∥a∥ denotes the Euclidean norm of a vector, E{·} stands for the statistical expectation, A⨂B captures the Kronecker product among the matrices, A⊙B captures the element-wise product among the matrices, $A_{M\times N\times L}$ denotes a three-dimensional matrix of size M,N,L, size(A) captures the dimensions (rows and columns) of matrix A, n=find(A(i,:)≠0) returns the columns of the *i*-th row of A with non-zero elements, and |n| stands for the length of vector n.

## 2. Spectrum Sensing Methods

In this section, we will present the most common spectrum detection techniques where each user individually decides if some frequency band in the spectrum is available for use. In particular, we will focus on the most popular in wireless communications, such as Energy Detector (ED), Cyclo-stationary Feature Detection (CFD), Compressive Detection (CD), and Matched Filter and Waveform-based technique [13,14]. Furthermore, we will present recent works on spectrum sensing exploiting the capabilities of machine/deep learning. Additionally, the section concludes with cooperative approaches.

### 2.1. Traditional Methods

An energy detector is a non-coherent method that identifies the primary signal based on the detected energy. It is the most common method due to its low computational and implementation complexity. Because of its simplicity and non-requirements for prior knowledge of the primary signals, the energy detector is the most popular technique in cooperatives spectrum sensing. The signal is detected by comparing the output of the detector to a threshold, which depends on the noise level [15]. However, the detection time to achieve a specific detection probability may be high. In addition to, the detection performance is subject to noise power uncertainty. An energy detector cannot be used to distinguish between the primary and CR signals. As a result, CR users need to be strongly synchronized and not transmit to the quiet period [6,16].

Cyclo-stationary Feature Detection (CFD) exploits the periodicity of the received PU signal to identify its presence. It is a detection method that takes advantage of the cyclo-stationary characteristics of the received signal, which is usually integrated into (i) sinusoidal carrier frequencies, (ii) pulse sequences, (iii) signal periodicity, or (iv) its statistical properties, such as mean value and autocorrelation [17]. Thus, CFD can distinguish between a telecommunication signal and the noise due to robustness at noise uncertainty, and performs better than the energy detector at low SNR. Although this requires prior knowledge of the signal characteristics, CFD can distinguish CR transmissions from various PU signals. It eliminates the requirement to synchronize the energy detector in cooperative detection. A weakness of this method is the high computational cost and long detection time. Because of these issues, it is less common than energy detectors in cooperative detection.

In the case of spectrum underutilization, compressive sensing techniques for cognitive radio networks can be used to approximate and recover the detected spectrum, which facilitates the detection of sparse primary signals in the wideband spectrum [18]. Hence, the compressive detection techniques provide promising solutions for the fast recovery of wideband signals and facilitate wideband detection with acceptable computational complexity. In wideband cooperative compressive detection, SU nodes individually perform compressive detection and cooperatively estimate the wideband spectrum by exchanging spectrum estimates, and iteratively reach a collective decision by exchanging local decisions [19].

Matched Filter [20] is the optimal method of detecting PUs signals when the transmitted signal is known. The main advantage is the short required time for achieving a certain probability compared to other methods that are discussed. The required number of samples increases with O(1/SNR) for a given false alarm probability $P_{fa}$ to low SNR. However, this requires the SU to modulate the received signals. Therefore, this requires perfect knowledge of the signal characteristics of PUs, such as bandwidth, operating frequency, modulation type, pulse shape, and format framework [21].

Waveform-based is usually used in wireless systems with known standards to help synchronization, or for other purposes. Such standards include preambles, midambles, spread-out sequences, etc. The preamble is a sequence transmitted before each burst. The midamble is transmitted in the middle of a burst or a time slot. It uses the same model as the energy detector. In the presence of a known pattern, detection can be performed by correlating the received signal with a known copy of it. This method only applies to systems with known signal patterns and is called coherent detection. This method is more reliable, has a better time convergence, and the performance of the algorithm increases with the length of the standard signal [21].

### 2.2. Recent Methods and Motivation

Apart from traditional spectrum sensing methods, researchers have turned to machine/deep learning [22], and Reinforcement and Federated Learning for efficient spectrum sensing. A reliable machine learning-based spectrum sensing in CR networks is presented in [23]. Wang et al. surveyed dynamic spectrum allocation based on reinforcement learning algorithms in cognitive radio networks [24]. Additionally, in [25], it is suggested that a machine-learning-based opportunistic spectrum access approach exploits multi-armed bandit (a powerful reinforcement learning tool) and matching theory. In [26], machine learning techniques (support vector machine (SVM), random forest (RF), and K-nearest neighbors (KNN)) are used to detect a PU in Mobile CR networks.

Classical local techniques can also be part of a cooperative framework for spectrum sensing, which is usually employed to increase the detection reliability in either a centralized (e.g., Fusion Center), decentralized, or relay-assisted manner [27,28,29]. In [30,31], cooperative spectrum sensing methods are suggested based on an energy detector. Alternative methods perform spectrum sensing in CR networks based on adaptive LMS-based distributed cooperative methods [12,32,33]. Recently, in [34], spectrum sensing is solved via federated learning, which is exploited for data distribution and model training over many devices. The proposed method creates a common deep learning model for each user group in the iterative process.

In the era of 5G networks, a high trend toward distributed and cooperative schemes has emerged for important operations in the physical layer, with some remarkable examples being spectrum sensing [35], beamforming [36], and channel estimation in the mmWave spectrum [37,38]. Due to the persistent increase in smart devices, and the need for high data rates with low latency, 5G and beyond networks have become more important than ever. Essential technology for its successful implementation is direct Device-to-Device (D2D) communication (e.g., for extending capacity and enabling scalability) with Internet-of-Things (IoT) being a characteristic short-range application that will highly benefit from the capabilities of 5G networks. Focusing on spectrum sensing in a 5G IoT network, geographically distributed SUs can sense multiple channels owned by PUs and decide via cooperation on available spectrum holes using learning algorithms (i.e., centralized and distributed).

In this study, distributed cooperative spectrum sensing will concern us, since such schemes are adaptable and more robust against changes in topology, especially in mobile and scalable IoT systems [39]. Focusing on a mobile CR network with overlapping spectrum PUs, a Bayesian machine learning approach for collaborative spectrum sensing has been suggested in [40]. In a more recent study [41], the authors, considering clusters of unmanned aerial vehicles, elaborated a diffusion-based distributed cooperative spectrum sensing approach with adaptive weights, assuming a binary state model to describe channel occupancy.

In the same direction, this study focuses on the problem of multi-task [42] parameter estimation, assuming varying spectrum sensing and adopting a distributed diffusion-based approach from a well-established family of techniques, which are preferable in wireless sensor networks due to lower communication overhead and more improved performance, compared with the centralized and non-cooperative strategies. In particular, we exploit the distributed diffusion method ATC to cooperatively estimate the aggregated spectrum, assuming a more complex scenario of three PUs with overlapping spectrums. Hence, multiple clusters of users are derived, each of which relates to a common interest frequency band. According to [11], a multi-task CR network is formulated. Initially, we evaluate the efficiency of ATC, assuming that the spectrum of PUs is varying (i.e., we cease the use of some frequency bands) and that secondary users are static. Finally, an interesting aspect of this work constitutes the performance evaluation of the same method in the case where one SU node is mobile.

## 3. Materials and Methods

In this section, we will present a description of the system model that approximates the total power spectrum in a specific SU node, the main aspects of the adopted solution method, and the definition of the necessary structures for the selected case study.

### 3.1. System Model

We consider *Q* PUs and *K* SUs. The power spectrum emitted by each PU can be captured as a linear combination of some basis functions. Here, we choose Gaussian basis functions. Each SU, through spectrum sensing, essentially detects the total spectrum from all of the PUs of the area. The power spectrum from PU *q* is written as [43,44]

(1) $$\begin{array}{cc} S_{q} & =\sum_{m=1}^{B}b_{qm}f_{m}\left(e^{j\omega }\right)\\ \; & =f_{\omega }w_{q},q=1,2,\ldots ,Q \end{array}$$

where $f_{m}\left(e^{j\omega }\right)=e^{-\frac{{\left(\omega -\omega_{m}\right)}^{2}}{2\sigma_{m}^{2}}}$ and parameters $\omega_{m},\sigma_{m}$ are the central frequency and standard deviation (these parameters are part of the system design and thus a priori known), $f_{\omega }=\left[\begin{array}{c} f_{1}\left(e^{j\omega }\right),f_{2}\left(e^{j\omega }\right),f_{3}\left(e^{j\omega }\right),\ldots ,f_{B}\left(e^{j\omega }\right) \end{array}\right]$ is a vector with the basis functions, scalars $\left\{b_{qm}\right\}$ denote the coefficients of the basis expansion for user *q*, and $w_{q}={\left[b_{q_{1}},b_{q_{2}},\ldots ,b_{q_{B}}\right]}^{T}$ is the vector with the factors involved in the linear combination of the basis functions. The representation in (1) can well approximate an essential part of the power spectrum if *B* is sufficiently large.

The power spectrum that a SU *k* detects by PU *q* is subjected to attenuation due to the propagation path loss denoted as $p_{qk}$. Path loss coefficients are known and defined in advance in a training stage between PUs with each SU. Training is usually repeated at regular intervals because the coefficients may change (slowly) in time due to node movement. When the transmitted spectrum travels from the PU to the SU, then the power spectrum that is measured by the receiver of the SU *k* is $p_{qk}S_{q}\left(e^{j\omega }\right)$.

Hence, the total power spectrum from all PUs at SU *k* is written as

(2) $$\begin{array}{cc} S_{k}^{t} & =\sum_{q=1}^{Q}p_{qk}S_{q}\left(e^{j\omega }\right)+\sigma_{k}^{2}\\ \; & =\sum_{q=1}^{Q}p_{qk}f_{\omega }w_{q}+\sigma_{k}^{2}\\ \; & =u_{k,\omega }w_{k}^{o}+\sigma_{k}^{2} \end{array}$$

where $w_{k}^{o}={\left[\begin{array}{c} w_{1}^{T},w_{2}^{T}\ldots w_{Q}^{T} \end{array}\right]}^{T}$ (Q·B×1) and $u_{k,\omega }=p_{kq}⊗f_{\omega }$ (1×Q·B) and $\sigma_{k}^{2}$ the receiver noise. Notice that $w_{q}^{T}$ stands for the $\{b_{qm}\}s$ involved in the power spectrum composition of PU *q*; thus, $w_{k}^{o}$ concatenates the $\{b_{qm}\}s$ of all PUs *Q*.

**Figure 1:**
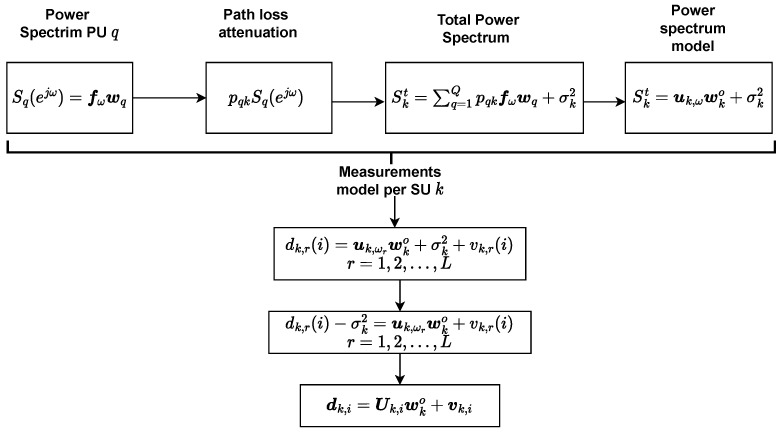
Processing steps involved in the system model.

At each time instant *i*, the SU *k* observes the received power spectrum in a discrete frequency grid $\left\{\omega_{r}\right\}$, in the interval [0,π] under the measurement and/or model noise $v_{k,r}$ with zero mean and covariance matrix $R_{v_{k}}$ of dimensions L×L:

(3) $$d_{k,r}\left(i\right)=u_{k,\omega_{r}}w_{k}^{o}+\sigma_{k}^{2}+v_{k,r},r=1,2,\ldots ,L$$

(4) $$d_{k,i}=\left[\begin{array}{c} d_{k,1}\left(i\right)-\sigma_{k}^{2}\\ d_{k,2}\left(i\right)-\sigma_{k}^{2}\\ \vdots \\ d_{k,L-1}\left(i\right)-\sigma_{k}^{2}\\ d_{k,L}\left(i\right)-\sigma_{k}^{2} \end{array}\right],v_{k,i}=\left[\begin{array}{c} v_{k,1}\left(i\right)\\ v_{k,2}\left(i\right)\\ \vdots \\ v_{k,L-1}\left(i\right)\\ v_{k,L}\left(i\right) \end{array}\right]$$

(5) $$U_{k,i}=\left[\begin{array}{c} u_{k,\omega_{1}}\\ u_{k,\omega_{2}}\\ \vdots \\ u_{k,\omega_{L-1}}\\ u_{k,\omega_{L}} \end{array}\right]=\left[\begin{array}{c} p_{k,i}⊗f_{\omega_{1}}\\ p_{k,i}⊗f_{\omega_{2}}\\ \vdots \\ p_{k,i}⊗f_{\omega_{L-1}}\\ p_{k,i}⊗f_{\omega_{L}} \end{array}\right]\left(L\times Q\cdot B\right)$$

(6) $$d_{k,i}=U_{k,i}w_{k}^{o}+v_{k,i}$$

where $v_{k}$ is the measurement and/or model noise with zero mean and covariance matrix $R_{v_{k}}$ of dimensions L×L. We have taken measurements at *L* different frequencies, and for this reason, the matrix has *L* rows. As a result, in (6), a linear model is obtained for estimating the parameters of interest in $w_{k}^{o}$. All of the considered processing steps are also illustrated in Figure 1.

The path loss factor is approximated according to the formula

(7) $$p_{qk,i}={\left(\frac{d_{qk,i}}{d_{0}}\right)}^{-n}$$

where $d_{qk,i}$ is the Euclidean distance of SU *k* to PU *q* at *i*, $d_{0}$ is the reference distance which is $d_{0}=1$, and *n* models the attenuation environment in the network. Hence, the path loss values between SU *k* and the *Q* PUs is captured in

(8) $$p_{k,i}=\left[\begin{array}{c} p_{1k,i},p_{2k,i},p_{3k,i},\ldots ,p_{Qk,i} \end{array}\right].$$

In the estimation of $p_{k,i}$, we consider a respective measurement Gaussian noise of zero mean and standard deviation $\sigma_{p}$; thus, ${\hat{p}}_{k,i}=p_{k,i}+n_{k}$. When the SU *k* moves, the values of $p_{k}$ change as its distance from the PUs changes, and consequently its values.

To estimate the spectrum, it is sufficient to estimate the parameters vector that multiplies the basis functions. By relying on the network data $\left\{d_{k,i},U_{k,i}\right\}$, we treat the problem as a parameter estimation of multiple interests and assume cooperation among the nodes to process the data in a distributed fashion based on a diffusion strategy, e.g., Adapt-Then-Combine (ATC). The aforementioned strategy well approximates the centralized solution when all nodes want to estimate the same vector of parameters [12].

Each vector ${\left\{w_{k}^{o}\right\}}_{k=1}^{K}$ might consist of parameters of global interest to the whole network, parameters of common interest to subsets of nodes including node *k*, and parameters of local interest for node *k*. In particular, subsets of parameters in $w_{k}^{o}$ may account for:

- One global parameter vector related to the frequency band in the power spectrum of all PUs, which affects all SUs in the network.
- *J* common parameter vectors associated with frequency bands in the power spectrum of PUs that affect specific subgroups of nodes with partially or fully overlapped common interests.

Finally, in this study, we have not considered parameters of local interest.

In this way, considering a scenario where there are *J* different subsets of common parameters, the observation model provided in (6) can be reformulated as

(9) $$d_{k,i}=U_{k_{g},i}w_{g}^{o}+\sum_{j\in I_{k}}U_{k_{c_{j}},i}\varsigma_{k,j}^{o}+v_{k,i},$$

where each node aims to solve through cooperation the following optimization problem

(10) $$\arg\min\sum_{k=1}^{K}E\{∥d_{k,i}-U_{k_{g},i}w_{g}-\sum_{j\in I_{k}}U_{k_{c_{j}},i}\varsigma_{k,j}{∥}^{2}\}$$

with respect to $w_{g},\varsigma_{1},\varsigma_{2},\ldots ,\varsigma_{J}$, where $I_{k}$ is an ordered set of indices *j* associated with the vectors $\varsigma_{j}$ that are of interest to node *k*, $U_{k_{g}}$, $U_{k_{c_{j}}}$ are matrices of dimensions $L\times M_{g}$, $L\times M_{c_{j}}$ that consist of the columns of $U_{k,i}$ associated with the $w_{g}$ and the $\varsigma_{k,j}$, respectively.

Some statistical independence assumptions [12] on the data are the following:

- $v_{k,i}$ is temporally and spatially white noise, whose covariance matrix is $R_{v_{k},i}=E\left\{v_{k,i}^{H}v_{k,i}\right\}$, and which is independent of $U_{k,i}$ for all *k* and *i*, with k∈{1,2,…,K} and i>0;
- $U_{k,i}$ is independent of $U_{k,j}$, with i,j>0 and i≠j (temporal independence);
- $U_{k,i}$ is independent of $U_{m,i}$, with k,m∈{1,2,…,K} and k≠m (spatial independence refers to different SUs);
- $U_{k_{g},i}$, $U_{k_{c_{j}},i}$ are independent for all k∈{1,2,…,K} and j∈{1,2,…,J} (independence among the global and common parameter vectors).

Under the assumption that all regressors $U_{k,i}$ are spatially and temporally independent, the unbiased asymptotic convergence of the diffusion-based ATC LMS algorithm with the previous combiners is satisfied for any initial condition, and any choice of the combiners, if step size μ takes values that are sufficiently small and that satisfy the inequality relation $0<\mu_{k}<2/\lambda_{max}(R\left(U_{k}\right)$, k=1,2…,K, where $\lambda_{max}$ is the maximum eigenvalue of the autocorrelation matrix $R(U_{k})$ of the transformed measurements’ model in (9).

### 3.2. Distributed Solution: ATC Diffusion-Based LMS

**Figure 2:**
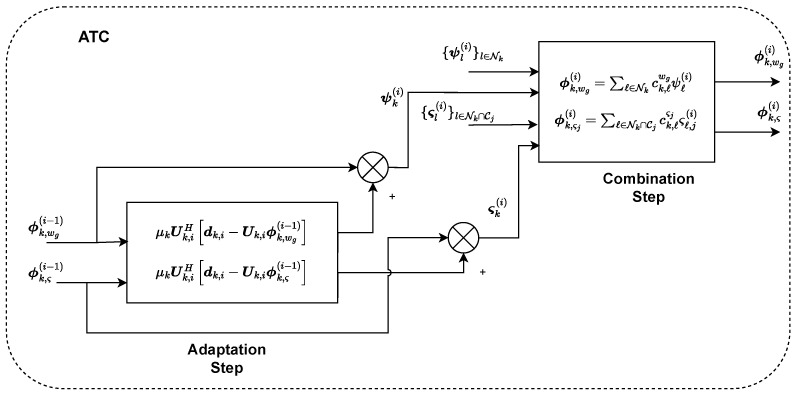
Illustration of Adapt-Then-Combine method at SU node *k*.

Distributed and adaptive schemes are usually adopted to improve energy efficiency, robustness, and scalability. In this study, we will exploit the diffusion method ATC, which consists of an adaptation and a combination step [12]. In the following, we will describe the main steps of the method, which are also illustrated in Figure 2. Before presenting ATC, a list of useful designations is shown in Table 1. The main steps of the ATC algorithm are the following:

- Assume $\phi_{k,w_{g}}^{\left(0\right)},{\left\{\phi_{k,\varsigma_{j}}^{\left(0\right)}\right\}}_{j\in I_{k}}$ at each node k∈{1,2,…,K}.
- For the estimation of $w_{g}^{o}$ and any $\varsigma_{j}^{o}$, choose K×K combination matrices $C^{w}$ and $C^{\varsigma_{j}}$ whose elements in each row *k*, i.e., ${\left\{c_{k,\ell }^{w_{g}}\right\}}_{\ell =1}^{K}$ and ${\left\{c_{k,\ell }^{\varsigma_{j}}\right\}}_{\ell =1}^{K}$, satisfy $c_{k,\ell }^{w_{g}}=0\;\mathrm{if}\;\ell \notin N_{k}$ and $\sum_{\ell \in N_{k}}c_{k,\ell }^{w_{g}}=1$, $c_{k,\ell }^{\varsigma_{j}}=0\;\mathrm{if}\;\ell \notin N_{k}\cap C_{j}$ and $\sum_{\ell \in N_{k}\cap C_{j}}c_{k,\ell }^{\varsigma_{j}}=1$, respectively.

Adaptation step at iteration *i*

(11) $$\left[\begin{array}{c} \psi_{k}^{\left(i\right)}\\ \varsigma_{k}^{\left(i\right)} \end{array}\right]=\left[\begin{array}{c} \phi_{k,w_{g}}^{\left(i-1\right)}\\ \phi_{k,\varsigma }^{\left(i-1\right)} \end{array}\right]+\mu_{k}U_{k,i}^{H}\left[d_{k,i}-U_{k,i}\left[\begin{array}{c} \phi_{k,w_{g}}^{\left(i-1\right)}\\ \phi_{k,\varsigma }^{\left(i-1\right)} \end{array}\right]\right]$$

Combination step at iteration *i*

(12) $$\phi_{k,w_{g}}^{\left(i\right)}=\sum_{\ell \in N_{k}}c_{k,\ell }^{w_{g}}\psi_{\ell }^{\left(i\right)},\phi_{k,\varsigma_{j}}^{\left(i\right)}=\sum_{\ell \in N_{k}\cap C_{j}}c_{k,\ell }^{\varsigma_{j}}\varsigma_{\ell ,j}^{\left(i\right)}$$

for each $j\in I_{k}$, $\varsigma_{k}^{\left(i\right)}=\mathrm{col}\left\{{\left\{\varsigma_{k,j}^{\left(i\right)}\right\}}_{j\in I_{k}}\right\}$ and $\phi_{k,\varsigma }^{\left(i\right)}=\mathrm{col}\left\{{\left\{\phi_{k,\varsigma_{j}}^{\left(i\right)}\right\}}_{j\in I_{k}}\right\}$. Once the algorithm terminates, $\phi_{k,w_{g}}$ and $\phi_{k,\varsigma_{j}}$s will be the estimates of the desired $w_{g}^{o}$ and $\varsigma_{j}^{o}$s. Assuming the clique topology, i.e., $|N_{k}\cap C_{j}\left|=\right|C_{j}|$ for all $k\in C_{j}$, the uniform combination rule creates the following combination weights:

(13) $$\begin{array}{cc} c_{k,l}^{w_{g}} & =\frac{1}{|N_{k}|}\\ c_{k,l}^{\varsigma_{j}} & =\frac{1}{|N_{k}\cap C_{j}|} \end{array}$$

The combination weights may be adaptive. Specifically, the following adaptive weighting mechanism is adopted,

(14) $$\begin{array}{cc} \gamma_{k,\ell }\left(i\right) & =\left(1-\nu \right)\gamma_{k,\ell }\left(i-1\right)+\nu {∥\psi_{\ell }^{\left(i\right)}-\phi_{k,w_{g}}^{\left(i-1\right)}∥}^{2}\\ \delta_{k,\ell }\left(i\right) & =\left(1-\nu \right)\delta_{k,\ell }\left(i-1\right)+\nu {∥\varsigma_{\ell }^{\left(i\right)}-\phi_{k,\varsigma }^{\left(i-1\right)}∥}^{2}, \end{array}$$

where *v* is a small positive factor in [0,1] and $\gamma_{k,\ell },\delta_{k,\ell }$ are the variances in the estimation of the global and common interest parameters. Then, the weights corresponding to both global and common parameter estimation processes are computed according to

(15) $$c_{k,\ell }^{w_{g}}\left(i\right)=\frac{\gamma_{k,\ell }^{-1}\left(i\right)}{\sum_{m\in N_{k}}\gamma_{k,m}^{-1}\left(i\right)},$$

(16) $$c_{k,\ell }^{\varsigma_{j}}\left(i\right)=\frac{\delta_{k,\ell }^{-1}\left(i\right)}{\sum_{m\in N_{k}\cap C_{j}}\delta_{k,m}^{-1}\left(i\right)}.$$

### 3.3. Case Study: Cognitive Radio Network with Three PUs of Overlapping Frequency Spectrum

**Figure 3:**
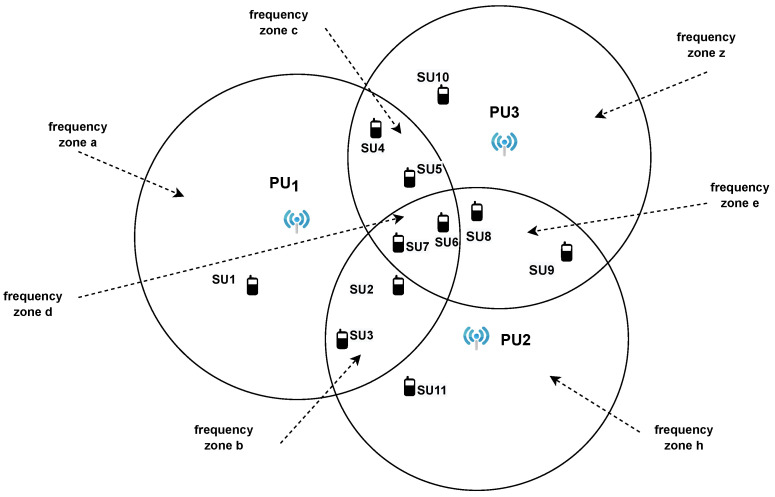
Cognitive radio network of three PUs with overlapping spectrum.

**Figure 4:**
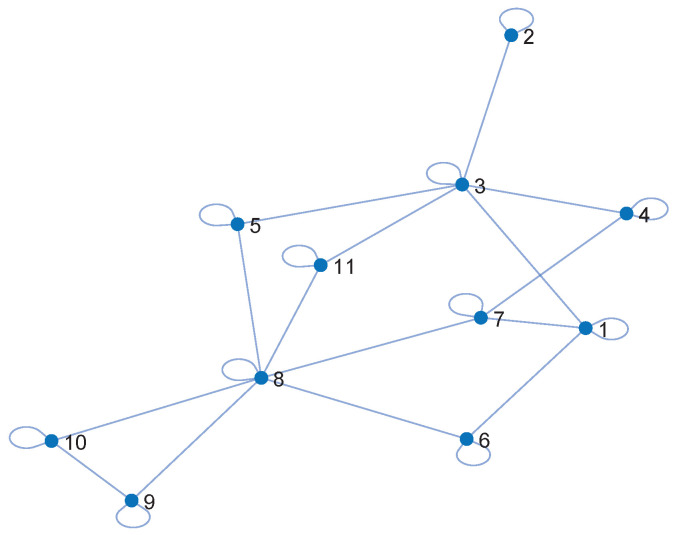
An overview of the adjacent matrix.

We have considered a network of Q=3 PUs with an overlapping power spectrum, as illustrated in Figure 3, and a set of K=11 geographically distributed SUs that communicate via a connected network, as shown in Figure 4. The nodes are interconnected by a graph G(V,E), where V={1,2,…,K} is the set of nodes and E={(i,j): Node i∈V is connected with node j∈V}.

In the network, there is one global interest frequency band *d* and J=6 common interest frequency bands denoted as a,b,c,e,z,h, where $PU_{1}=\left\{a,b,c,d\right\}$, $PU_{2}=\left\{b,d,e,h\right\}$ and $PU_{3}=\left\{c,d,e,z\right\}$.

Each of the following sets includes the nodes interested in the corresponding frequency band, and thus, the parameters of $w_{k}^{o}$ involved in the spectrum approximation. Based on this concept, we assume that there are the following sets:

- Frequency zone *a*: $C_{1}=\left[1,2,3,4,5,6,7\right]$
- Frequency zone *b*: $C_{2}=\left[1,2,3,4,5,6,7,8,9,11\right]$
- Frequency zone *c*: $C_{3}=\left[1,2,3,4,5,6,7,8,9,10\right]$
- Frequency zone *e*: $C_{4}=\left[2,3,4,5,6,7,8,9,10,11\right]$
- Frequency zone *z*: $C_{5}=\left[4,5,6,7,8,9,10\right]$
- Frequency zone *h*: $C_{6}=\left[2,3,4,5,8,9,11\right]$

According to these sets, SU1 is located at the coverage area of PU1 and is interested in frequency bands a,b,c,d. SU4 is in the range of PU1, PU2, and PU3, and is interested in all frequency bands. SU2 is in the coverage of PU1 and PU2 and is interested in all zones except for *z*. Based on the common interest sets $C_{j},j=1,2,\ldots ,J$ with J=6, we construct a binary matrix whose columns refer to the number of global and common frequency bands or interest parameter vectors, and its rows capture the SUs in the network. Its size is K×(J+1) and is defined as $C\left(k,j\right)=\left\{\begin{array}{cc} 1 & \mathrm{if}k\in C_{j}\\ 0 & elsewhere \end{array}\right.$∀j∈1,2,…,J+1 and k∈1,2,…,K. Note that the first column of the matrix refers to the frequency band *d* that all SU nodes are interested in, while the remaining relate to the common interest bands a,b,c,e,h,z. More specifically, this matrix is as follows:

(17) $$C=\left[\begin{array}{ccccccc} 1 & 1 & 1 & 1 & 0 & 0 & 0\\ 1 & 1 & 1 & 1 & 1 & 0 & 1\\ 1 & 1 & 1 & 1 & 1 & 0 & 1\\ 1 & 1 & 1 & 1 & 1 & 1 & 1\\ 1 & 1 & 1 & 1 & 1 & 1 & 1\\ 1 & 1 & 1 & 1 & 1 & 1 & 0\\ 1 & 1 & 1 & 1 & 1 & 1 & 0\\ 1 & 0 & 1 & 1 & 1 & 1 & 1\\ 1 & 0 & 0 & 1 & 1 & 1 & 0\\ 1 & 0 & 1 & 0 & 1 & 0 & 1 \end{array}\right]$$

Moreover, we consider that for each of the frequency bands of common interest, a parameters’ vector of size $M_{c_{j}}\times 1$ should be estimated, while for the global interest frequency band, one parameters vector of size $M_{g}\times 1$ should be estimated. We create the matrix $C_{k}^{aug}$ of size $L\times (M_{g}+\sum_{j=1}^{J}M_{c_{j}})$ based on the interests matrix C. At each row of the matrix C, the first column element is duplicated $M_{g}$ times, and each one of the rest elements is duplicated $M_{c_{j}}$ times. Then, each row is duplicated *L* times. Hence, the augmented matrix $C_{k}^{aug}$ is created and the "correct" input matrix at ATC is $U_{aug}=C_{k}^{aug}U_{k,i}$ (point by point multiplication) at SU node *k* and time instant *i*. Essentially, the $C_{k}^{aug}$ fills with zeros the rows and columns of $U_{k,i}$ that correspond to those common parameters, which are out of the interest of node *k*. The acquired $U_{aug}$ will multiply the augmented parameters vector $w_{k}={\left[{w_{g}}^{T}\;\varsigma_{1}^{T}\;\varsigma_{2}^{T}\ldots \varsigma_{J}^{T}\right]}^{T}\;\left(M_{k}\times 1\right)$, which, based on (6), is used to collect the measurements $d_{k,i}$.

The performance evaluation of the ATC method is based on Mean Square Deviation (MSD), assuming different combination rules, i.e., a uniform clique and a relative variance with adaptive weights. The MSD is defined as

(18) $$\begin{array}{cc} {\mathrm{MSD}}_{k} & =\lim_{i\to \infty }E\left\{{∥w_{k}^{o}-{\hat{w}}_{k,i}∥}^{2}\right\}\\ \mathrm{MSD} & =\frac{1}{K}\sum_{k=1}^{K}{\mathrm{MSD}}_{k}, \end{array}$$

where $w_{k}^{o},{\hat{w}}_{k,i}$ are respectively the optimal and estimated parameter vectors that contain in concatenate form the $M_{g}$ global and those $M_{c_{j}}$ common interest parameters that concern node *k*. An algorithmic overview of the above processing steps is outlined in Algorithms 1–3. More specifically, Algorithm 1 is utilized to generate the data that will be used as input to Algorithm 3, which estimates the parameters vector and the MSD for all users in the network.

Additionally, this algorithm exploits internally Algorithm 2, which implements the Equations (13)–(16) in order to create the matrices $C^{g},C^{c_{j}}$ involved in the combination step of global and common (uniform and adaptive, respectively). If we set t=1 (global) and t=2 (common) in the Rule functions inside Algorithm 3, the results correspond to the uniform weights combination rule.
**Algorithm 1:** Data generation 
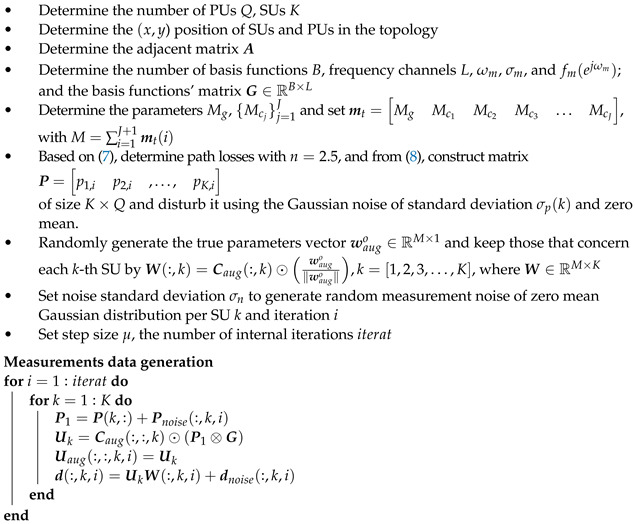


**Algorithm 2:** Rule 
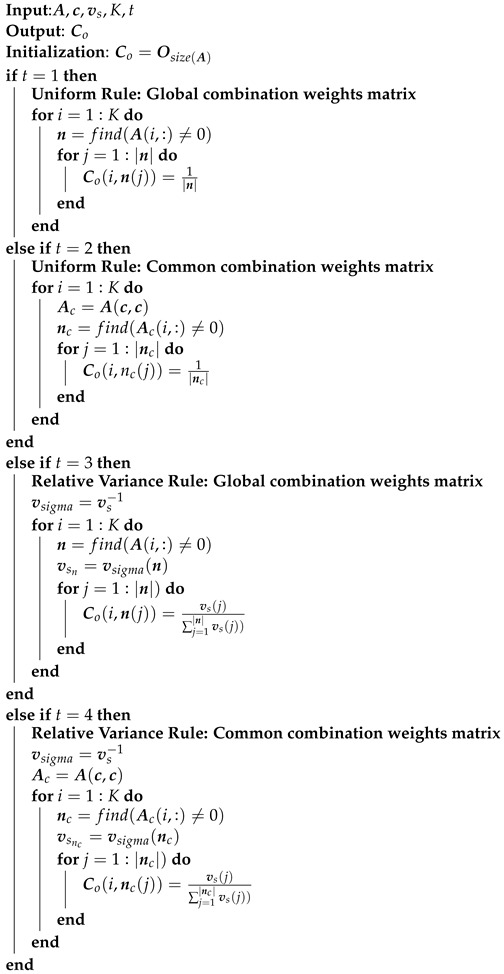


**Algorithm 3:** Diffusion-based Distributed Cooperative Method, Adapt-Then-Combine 
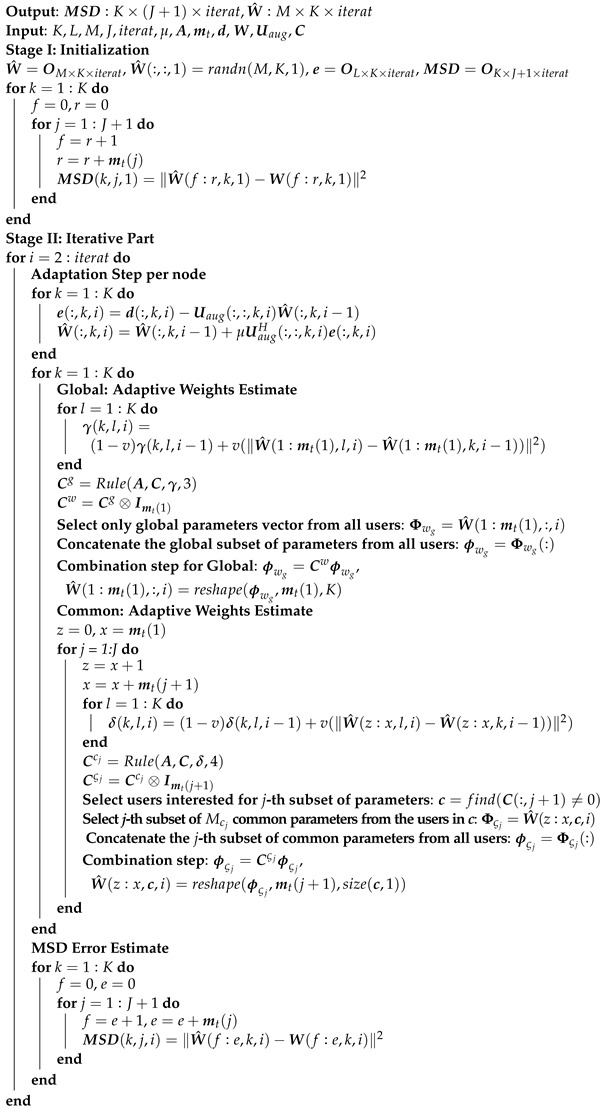


## 4. Results and Discussion

In this section, we present the results of the ATC algorithm performance evaluation, which is used to estimate the $Q\cdot B=M_{k}=M_{g}+\sum_{j\in I_{k}}M_{c_{j}}$ parameters that approximate the total power spectrum emitted by the three PUs. Here, we assume that $M_{1}=M_{2}=\ldots =M_{K}=M$. The problem is translated into a multiple interests (global and common) parameter estimation problem.

**Figure 5:**
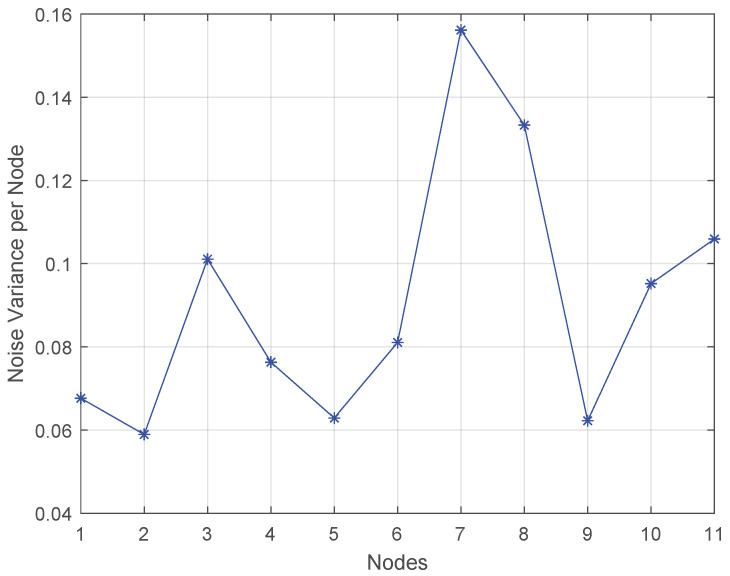
Noise variance per node.

The performance of the adopted method was evaluated using Monte Carlo simulations with 50 independent runs [45]. We simulated a network consisting of Q=3 PUs and K=11 SUs, as illustrated in Figure 3. Additionally, we have considered B=16 Gaussian basis functions of amplitude normalized to one and standard deviation $\sigma_{m}=0.05$. These functions are involved in (2), and especially, in the creation of $U_{k,i}$ in the linear model of (6). Furthermore, each SU scans L=50 channels over the normalized frequency axis between 0 and 1. The noise $v_{k,i}$ in (6) or (9) is zero-mean Gaussian with a standard deviation varying between 0.04 and 0.16 for different *k*. Similarly, the additive noise $n_{k}$ in path loss estimation is generated with $\sigma_{p}\in \left[0.03,1.25\right]$. In Figure 5, we illustrate the noise power of each SU node. The step-size $\mu_{k}$ of the LMS adaptation at each node is equal to 0.02∀k. Additionally, we selected $M_{g}=10,M_{c_{1}}=3,M_{c_{2}}=5,M_{c_{3}}=6,M_{c_{4}}=7,M_{c_{5}}=8,M_{c_{6}}=9$.

### 4.1. Static SU Nodes and Varying Spectrum

Here, the term varying spectrum is utilized to capture the case where the PUs ceases to use some of the frequent zones a,b,c,d,e,z,h. In this case, we aim to examine the behavior of the ATC algorithm by monitoring if the parameters’ estimation error has the desired behavior. SUs are not aware of these changes, but through the measurements $\left\{d_{k,i},U_{k,i}\right\}$ and the estimation error of the involved parameters that approximate the total power spectrum, they learn of the changes that occur in the broadcasting spectrum of the PUs. To evaluate the above conditions, we run the algorithm so that in different time intervals, the use of a particular frequency band is cancelled. The cancellation is achieved by setting zero values to the corresponding optimal parameters in the augmented vector $w_{k}$ which is used internally in the algorithm for estimating the MSD. Specifically, we selected to cancel, in specific time intervals, the use of the following parameter vectors:

- 15–20: $M_{g}$ parameters of $w_{g}$,
- 60–70: $M_{c_{1}}$ parameters of $\varsigma_{1}$,
- 110–120: $M_{c_{2}}$ parameters of $\varsigma_{2}$.

**Figure 6:**
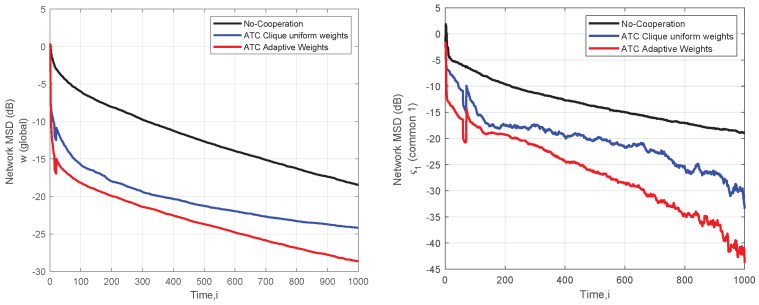

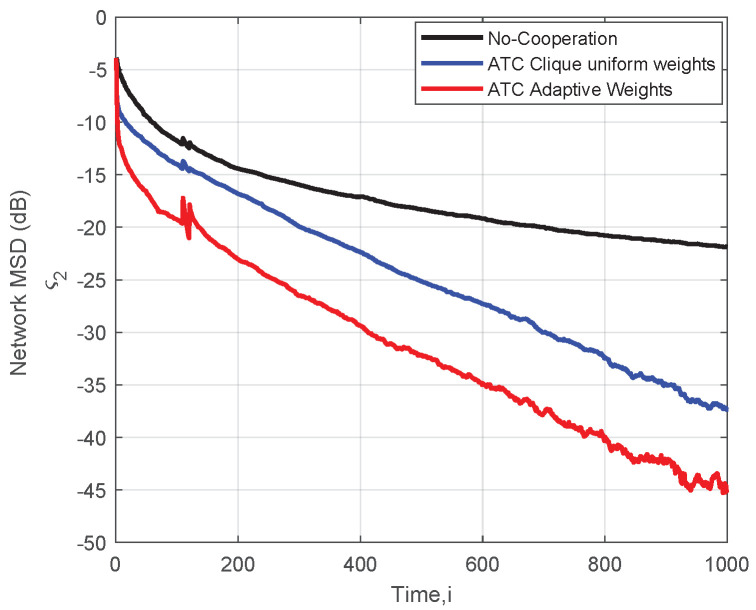
Network MSD (dB) for the *w* (global) and commons $\varsigma_{1},\varsigma_{2}$.

In Figure 6, we present the average performance of MSD error for the aforementioned interest vectors. As it is shown, the ATC method identifies this change in the sensing environment, which is captured in the MSD error of these parameter vectors. For comparison, we also plot the performance of the non-cooperative LMS algorithm, setting the adjacent matrix as $A=I_{K}$. The results verify the importance of cooperation in relation to individual estimation. Moreover, comparing the two methods for the estimation of the combination weights, the superiority of adaptive filtering is verified. The nodes working together to achieve spectrum estimation do not know in advance which neighboring nodes are affected by which model and which model affects their own data. A node with different interests may feed its neighbors with irrelevant data, that is, with incorrect estimates that alter their own. The nodes gradually adjust the weight they give to the estimates of that neighbor, which tends to zero, so the effect of its estimate becomes negligible. That is, the network is divided into clusters-groups according to the interests of the nodes. As we see, the collaboration between members of a group with the same interest leads to a better estimation of the parameters of interest. The weights’ values are a measure of trust that a node gives to its neighboring node.

Focusing on the convergence of the ATC, we observe that in the case of the global parameters vector, the algorithm starts to approach the steady state at iteration i=1000. However, much more iterations are needed in the case of the two common interest parameter vectors for the MSD to attain a steady state. Additionally, an essential performance difference occurs from the average individual estimate of all nodes. Observing the MSD of the two common interest parameter vectors, it is lower than the MSD of the global parameters.

### 4.2. Mobile CR Node

In this subsection, as a first approach, we evaluate the performance of ATC, assuming that there is one mobile SU node in the network. A moving SU changes position (x,y). However, this change may not necessarily vary the frequency bands of interest and the neighbors. If changes occur, this implies that the corresponding structures of the ATC algorithm should be updated. These structures relate to the path loss coefficients between the mobile SU and the three PUs because it is location-dependent, the adjacency matrix A and the interests’ matrix C. To check the area in which the moving node is located, we measure the node’s Euclidean distances from the three PUs and compare them with the corresponding transmission range of the PUs. Let’s assume that the distances are $d_{1},d_{2}$, and $d_{3}$, respectively, and the transmission ranges are $R_{1},R_{2}$, and $R_{3}$. For the specific experiment, we considered $R_{1}=150,R_{2}=165$, and $R_{3}=170$. Based on Figure 3, we formulated the rules presented in Table 2, which helps us to update the interests’ matrix C and the structures that depend on it. Moreover, in Table 3, we assume the characteristic points of the SU1 trajectory and the corresponding frequency zones of interest derived from Table 2 under the selected values for the parameters $R_{1},R_{2},R_{3}$.

**Figure 7:**
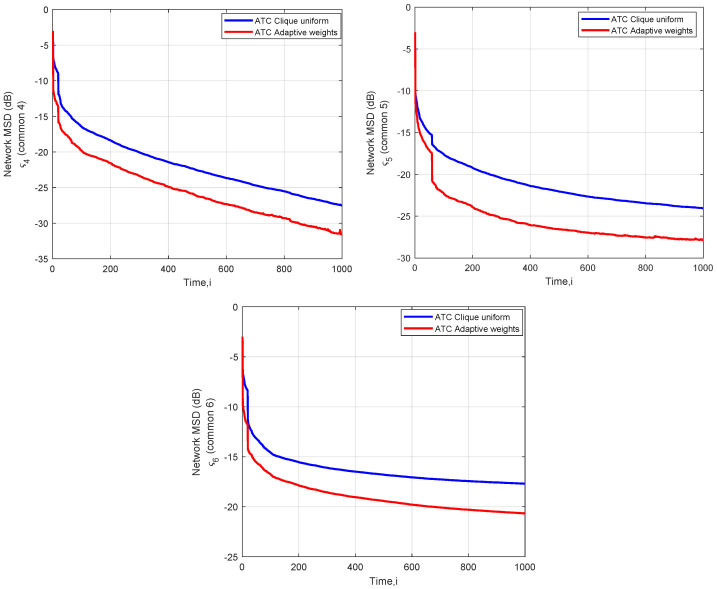
Network MSD (dB) for the common interest vectors $\varsigma_{4},\varsigma_{5},\varsigma_{6}$.

Now, let us focus on Figure 7, which captures the MSD curves of those parameter vectors that correspond to the frequency zones (i.e., *e*, *z*, *h*) that started to be in the interests of the SU1 node and that relate to the common interest vectors $\varsigma_{4},\varsigma_{5},\varsigma_{6}$. Focusing on $\varsigma_{4},\varsigma_{5},\varsigma_{6}$ and the convergence of the ATC, we see that in the case of $\varsigma_{5},\varsigma_{6}$ the algorithm approaches the steady state earlier, at iteration i=500. However, more iterations are needed (at least i=1000) in the case of $\varsigma_{4}$ to attain the steady state. An essential performance distance is captured between the adaptive and the uniform weighting methods, where the adaptive weights win. Finally, we observe an abrupt fall in the MSD in the time instants 19 and 59, as indicated in Table 3, which shows that the algorithm identifies the new parameters of interest of SU1.

**Figure 8:**
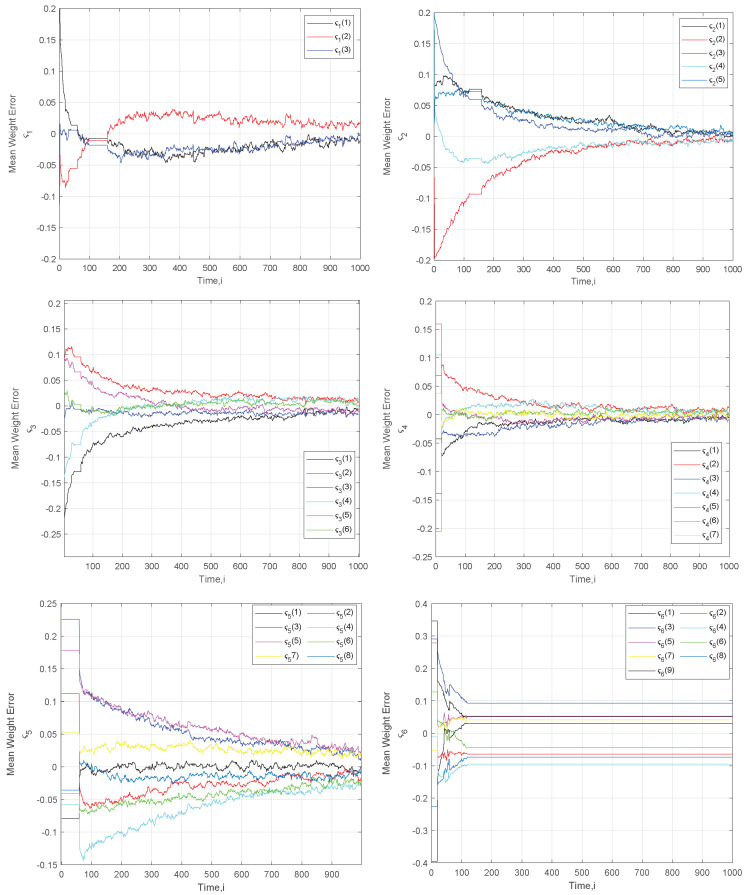
Mean Weight Error from the true parameters for the common vectors ${\left\{\varsigma_{j}\right\}}_{j=1}^{6}$.

Assuming the adaptive weights method in the combination part of the ATC, in Figure 8, we capture the mean weight error of the parameters of interest of the moving SU1, which as it moves, changes regions and frequency bands of interest. In each figure, we depict the evolution of the $M_{c_{j}}$ parameters estimation error. Observing Table 3, we see that the time intervals where the mean error curve is flat correspond to regions where the SU1 is not interested in the respective frequency band.

In concluding the evaluation part of the adopted ATC method, we see that the algorithm is able to identify the changes in the interests of a SU, which is reflected in the error curves. In either case, its performance remains intact, achieving low error values that attain zero.

## 5. Conclusions

In conclusion, the estimation of the total power spectrum of three PUs was investigated via the ATC diffusion algorithm, which was tackled as a multi-task problem, having promising results in each of the considered scenarios. We examined its efficiency for two different weighting rules for the coefficients in the combination step of the algorithm. In the case of the relative variance rule combined with the adaptive estimation filter, MSD achieved better performance than the uniform one. This is due to the fact that the noise profile of the nodes is derived based on the instant estimates of the parameters vector, both of them and their neighbors.

An open issue in the above scenarios could be the usage of more overlapping primary users, which would, however, eliminate the zone of global interest, and nodes would only estimate various common interest parameters. Additionally, a variation of the examined scenarios could concern the movement of more than one node, since, in practical scenarios, more SUs may be mobile.

Alternatively, we could determine the combination weights, taking into account only the variance of two communicating nodes and not of the whole neighborhood, and evaluate its benefits in the per node and network MSD in the adaptive combination weights. Our aim is to see the improvements to MSD by experimenting with the parameters involved in the ATC method and especially the step size μ, which is an important parameter that impacts on the convergence of the algorithm. Finally, it would be interesting to re-evaluate the above parameter estimation problem, if we were using the Combine-Then-Adapt or LMS-type incremental methods.

## Figures and Tables

**Table 1:** List of designations.

Notation	Description
*K*	Number of SU nodes
*Q*	Number of PU nodes
$C_{j}$	Set of SU nodes for common interest parameter vector j=1,2,…,J
A	Adjacent matrix
$C^{g}$	K×K keeps the global weights $c_{k,l}^{w}$
$C^{w}$	$KM_{g}\times KM_{g}$ $C^{w}=C^{g}⊗I_{M_{g}}$
$C^{c_{j}}$	$|C_{j}\left|\times \right|C_{j}|$ keeps the common weights $c_{k,l}^{\varsigma_{j}}$
$C^{\varsigma_{j}}$	$M_{c_{j}}|C_{j}|\times M_{c_{j}}\left|C_{j}\right|$ $C^{\varsigma_{j}}=C^{c_{j}}⊗I_{M_{c_{j}}}$
$I_{M_{c_{j}}},I_{M_{g}}$	Identity matrix of size $M_{c_{j}},M_{g}$
$M_{g}$	Number of global interest parameters
$M_{c_{j}}$	Number of common interest parameters
$M_{k}\leq M_{g}+\sum_{j=1}^{J}M_{c_{j}}$	Maximum number of parameters of interest (global and common)

**Table 2:** Rules to determine the frequency zone of a mobile node.

Rules	Frequency Zone
$d_{1}<R_{1}$ & $d_{2}>R_{2}$ & $d_{3}>R_{3}$	*a*
$d_{1}<R_{1}$ & $d_{2}<R_{2}$ & $d_{3}>R_{3}$	*b*
$d_{1}<R_{1}$ & $d_{2}>R_{2}$ & $d_{3}<R_{3}$	*c*
$d_{1}<R_{1}$ & $d_{2}<R_{2}$ & $d_{3}<R_{3}$	*d*
$d_{1}>R_{1}$ & $d_{2}<R_{2}$ & $d_{3}<R_{3}$	*e*
$d_{1}>R_{1}$ & $d_{2}>R_{2}$ & $d_{3}<R_{3}$	*z*
$d_{1}>R_{1}$ & $d_{2}<R_{2}$ & $d_{3}>R_{3}$	*h*

**Table 3:** Mobile CR node positions and frequency zones.

Time Interval	Position	Frequency Zone
1–19	(−40,170)	*a,b,c,d*
20–39	(120,150)	*a,b,c,d,e,h*
40–59	(280,160)	*b,d,e,h*
60–79	(100,270)	*a,b,c,d,e,z,h*
80–99	(110,300)	*a,b,c,d,e,z,h*
100–119	(190,320)	*b,c,d,e,z,h*
120–139	(220,390)	*c,d,e,z*
140–159	(130,390)	*c,d,e,z*
160–500	(10,310)	*a,b,c,d,e,z*
